# CP7_E2alf oral vaccination confers partial protection against early classical swine fever virus challenge and interferes with pathogeny-related cytokine responses

**DOI:** 10.1186/1297-9716-44-9

**Published:** 2013-02-11

**Authors:** Patricia Renson, Mireille Le Dimna, André Keranflech, Roland Cariolet, Frank Koenen, Marie-Frédérique Le Potier

**Affiliations:** 1 Anses, Ploufragan/Plouzané laboratory, Swine Virology and Immunology Unit, BP53, 22440, Ploufragan, France; 2 Anses, Ploufragan/Plouzané laboratory, Pathogen-free Pig Breeding and Testing Facility, BP53, 22440, Ploufragan, France; 3 CODA-CERVA, Interactions and Surveillance, 99 Groeselenberg, B-1180, Brussels, Belgium

## Abstract

The conventional C-strain vaccine induces early protection against classical swine fever (CSF), but infected animals cannot be distinguished from vaccinated animals. The CP7_E2alf marker vaccine, a pestivirus chimera, could be a suitable substitute for C-strain vaccine to control CSF outbreaks. In this study, single oral applications of CP7_E2alf and C-strain vaccines were compared for their efficacy to induce protection against a CSF virus (CSFV) challenge with the moderately virulent Bas-Rhin isolate, in pigs as early as two days post-immunization. This work emphasizes the powerful potential of CP7_E2alf vaccine administered orally by a rapid onset of partial protection similar to that induced by the C-strain vaccine. Furthermore, our results revealed that both vaccinations attenuated the effects induced by CSFV on production of the pig major acute phase protein (PigMAP), IFN-α, IL-12, IL-10, and TGF-β1 cytokines. By this interference, several cytokines that may play a role in the pathogeny induced by moderately virulent CSFV strains were revealed. New hypotheses concerning the role of each of these cytokines in CSFV pathogeny are discussed. Our results also show that oral vaccination with either vaccine (CP7_E2alf or C-strain) enhanced CSFV–specific IgG2 production, compared to infection alone. Interestingly, despite the similar antibody profiles displayed by both vaccines post-challenge, the production of CSFV-specific IgG1 and neutralizing antibodies without challenge was lower with CP7_E2alf vaccination than with C-strain vaccination, suggesting a slight difference in the balance of adaptive immune responses between these vaccines.

## Introduction

Classical swine fever (CSF) is a highly contagious viral disease in swine that leads to important economic losses worldwide. The CSF virus (CSFV), a *Pestivirus* member of the *Flaviviridae* family, is a small enveloped RNA virus encoding four structural proteins and eight non-structural proteins. The E2 glycoprotein is the most immunogenic CSFV protein. The severity of clinical signs varies according to host parameters but is also dependent on the virulence of the viral strains [1]. Highly virulent (HV) strains cause an acute hemorrhagic form of the disease that induces marked immune suppression and high mortality, whereas moderately virulent (MV) strains induce either a sub-acute or a chronic form of the disease from which pigs may recover.

Most countries in the European Union (EU) are CSF-free, but the virus can periodically be reintroduced via wild boars which may constitute a reservoir. Most of the recently isolated European strains are MV strains belonging to the 2.3 genotype. Since the 1990s, a non-vaccination stamping-out policy has been enforced in the EU, leading to pre-emptive culling in affected countries [2]. Despite the availability and efficacy of the conventional C-strain vaccine (an attenuated live strain of CSFV) in disease prevention, vaccinated animals cannot be distinguished from infected animals by serological diagnosis. In some EU countries, the aim of oral vaccination campaigns is to eradicate the virus in wild boar populations by using baits containing the C-strain vaccine [3,4]. Thus, efficacy induced by administration both by the intramuscular route to domestic pigs and by oral route to wild boars would be an asset for a new vaccine.

Live marker vaccines against CSFV, based on viral vectors or chimeric pestiviruses, are the most promising option for rapid onset of protection, with both single dose and oral application possibilities [5]. The modified vaccine CP7_E2alf is a chimera of the bovine viral diarrhea virus expressing the CSFV E2 protein. It displays promising potency and induces similar effective protection against a CSFV challenge to the C-strain [6]. The CP7_E2alf vaccine behaves similarly to CSFV and uses the same primary replication site [7]. Moreover, the safety and efficiency of the CP7_E2alf vaccine in both intramuscular and oral application protocols has been demonstrated in pigs as well as wild boars, together with persistent immunity for 6 months post-vaccination [8-10]. The C-strain vaccine is able to induce full clinical protection and partial protection against an HV strain of CSFV 4 and 2 days post-vaccination, respectively [11]. The C-strain vaccine can also induce complete protection against a MV strain at 5 days post-vaccination and partial protection after 3 days [12]. The CP7_E2alf marker vaccine confers full clinical protection against an HV strain challenge 7 days post-intramuscular immunization or 14 days post-oral immunization [13].

Protection from the disease is mainly associated with the humoral immune response but CSFV-neutralizing antibodies usually appear about 2 weeks post-vaccination. Thus, the early efficacy of the C-strain vaccine indicates that other immune responses may also be involved, such as cell-mediated immunity with significant IFN-γ production in relation to protection [14]. CSFV infection is characterized by the enhanced release of IFN-α and proinflammatory cytokines, such as TNF-α, IL-6 and IL-1, which disturb the homeostasis of the cellular environment [15-17]. Conversely, vaccine combinations with human IFN-α or porcine IL-6 improve immunity to CSFV in pigs [18,19]. The respective role of cytokines in pathogeny or protection remains to be determined.

In this study, we compared the capacities of a single oral application of CP7_E2alf or C-strain vaccine to protect swine from a challenge with a MV CSFV field strain, as early as 2 days post-oral vaccination. The powerful potential of the CP7_E2alf marker vaccine as a substitute for C-strain vaccine was demonstrated. Mainly, the investigation of immune responses against early challenge by antibodies and cytokine profile-induced post-oral vaccination, suggested that the CP7_E2alf vaccine, like the C-strain vaccine, interferes with the cytokine responses induced by CSFV infection. Differences in adaptive immune responses induced between the C-strain vaccine and the CP7_E2alf vaccine were also suggested by antibody profiles.

## Materials and methods

### Vaccines and virus

RIEMSER^®^ Schweinepestvakzine baits (vaccine solution from the blister of baits used for the wild boar oral vaccination campaign) were used for C-strain immunization (Riemser Arzneimittel AG, Greifswald - Insel Riems, Germany). The CP7_E2alf vaccine [6] was produced by Pfizer (Pfizer Olot, La Vall de Bianya, Spain). The MV Bas-Rhin strain (genotype 2.3) used for the CSFV challenge was isolated from a wild boar in France in 2003 and propagated for 3 passages on porcine kidney cell line PK15.

### Animal experiment design

Nine-week-old specific-pathogen-free Large White pigs from our protected facilities were randomly assigned to six groups of five animals housed in six separate rooms. Two groups were orally vaccinated with a single protective dose of either C-strain vaccine or CP7_E2alf vaccine (minimum titre of 10^4.5^ TCID_50_/dose for C-strain and 10^5.5^ TCID_50_/dose for CP7_E2alf) and oro-nasally challenged two days later with the Bas-Rhin strain (10^6^ TCID_50_/pig). Two other groups were vaccinated with either C-strain vaccine or CP7_E2alf vaccine and one group was infected with the Bas-Rhin strain. One group was neither vaccinated nor infected and served as the control. For oral vaccination, a solution of either C-strain vaccine or CP7_E2alf vaccine was administered in the mouth using a 2 mL syringe without a needle. For oro-nasal infection, 2 mL of the Bas-Rhin viral solution was injected into each nostril and 1 mL was administered in the mouth using a 5 mL syringe without a needle.

Rectal temperature, weight gain and clinical score were monitored daily as previously described [20]. Blood samples were collected before vaccination (on day 0), at 5 days post-vaccination (dpv) (i.e. 3 days post-infection (dpi) or challenge) and then 2 or 3 times per week until 27 dpv (i.e. 25 dpi).

The animal experiment protocol was approved by the French national ethics committee ComEth Anses/ENVA/UPEC.

### Sample treatment

Blood lymphocyte counts were measured with a MS9 hematology analyzer (Melet Schloesing laboratoires, Osny, France). Serum samples were purified from coagulated blood samples by centrifugation at 3000 × *g* for 5 min.

### Virological tests

RNA was purified from whole blood using the RNeasy mini kit (Qiagen, Courtaboeuf, France). The CSFV genome was detected by performing real time RT-PCR (rRT-PCR) using the commercial TaqVet CSF kit (LSI, Lissieu, France) in which the primers are located in 5’NTR. Specific genome detection for either C-strain or CP7_E2alf vaccine was assessed by rRT-PCR as previously described [21]. Virus titration from blood was performed on PK15 cells, according to the OIE’s manual of diagnostic tests [22].

### Serological tests

CSFV-specific IgG1 and IgG2 were measured in serum by indirect ELISA. CSFV-infected cell extracts (Alfort 187 strain), prepared using a detergent solution containing 2% Octyl b-D-glucopyranoside, were applied to Maxisorp microplates (Nunc, Roskilde, Denmark), incubated overnight at RT and blocked for 1 h at RT with 10% FBS-2% milk-PBS. Thereafter, sera were incubated for 1 h at 37°C with either mouse anti-porcine IgG1 at 1:250 or IgG2 at 1:100 (AbD Serotec, Oxford, UK). For detection, rabbit anti-mouse IgG HRP-conjugated secondary antibody was added at 1:250 (DAKO, Glostrup, Denmark) for 30 min at 37°C and peroxidase activity was measured using TMB substrate (Sigma-Aldrich, St Louis, USA) at 450 nm.

CSFV-specific neutralizing antibodies (NAb) against the Bas-Rhin strain were assayed using the virus neutralization test on PK15 cells according to the OIE’s manual of diagnostic tests [22].

### Cytokine quantifications

Porcine cytokines were measured in serum using ELISA kits from Invitrogen for IL-18, IL-10, IL-4, IFN-γ and TNF-α (Life Technologies, Carlsbad, USA), from R&D systems for IL-1β, TGF-β1, IL-6 and IL-12 (R&D systems, Minneapolis, USA) and from Kitvia for pig major acute phase protein (pigMAP) (KitVia, Labarthe Inard, France). Porcine IFN-α was detected using an ELISA test, as previously described [17].

Porcine IL-10, IL-4 and IFN-γ gene expressions were assessed in unpurified cells from whole blood using real time RT-PCR performed using the SuperScript III Platinum One-Step Quantitative RT-PCR System (Life Technologies, Carlsbad, USA). β-actin was used as endogenous control to normalize the quantification of target genes. Previously published primers and probes were used for cytokines [23] and β-actin [24]. The relative expressions of target genes were calculated using the R = 2^–ΔΔCT^ equation.

### Statistical tests

The measured variation for a treated group was compared with that of the control group (statistical significance indicated in the figures) or another treated group and analyzed by Mann–Whitney test. Statistical tests were performed using Systat 9 software (Systat Software Inc., Point Richmond, USA), with the limit of significance set at *p* < 0.05. The error bars on the graphs represent the standard error (SE).

## Results

### CP7_E2alf vaccine attenuates clinical expression of the disease

**Figure 1:**
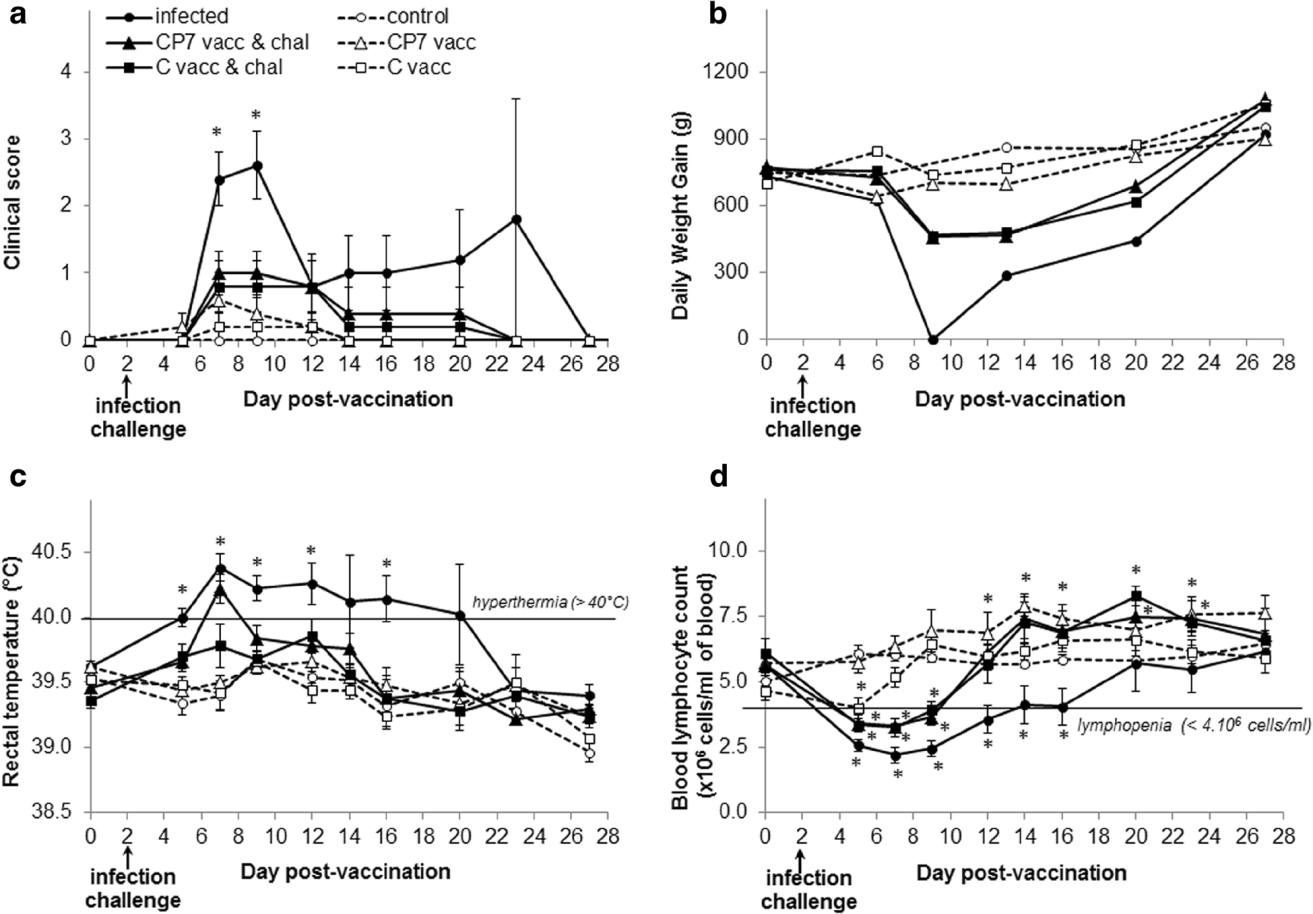
**Clinical parameters monitored in experimental groups.** (**a**) Clinical score, (**b**) Daily weight gain, (**c**) Rectal temperature and (**d**) Blood lymphocyte count. All data are reported as the mean (± SE) of results obtained for the 5 pigs in each group. *p < 0.05 compared each post-vaccination, post-challenge or post-infection value with the respective control value using the Mann–Whitney test. Dotted line with white triangles: CP7_E2alf vaccinated group (CP7 vacc); dotted line with white squares: C-strain vaccinated group (C vacc); dotted line with white circles: control group; full line with black triangles: CP7_E2alf vaccinated and challenged group (CP7 vacc & chal); full line with black squares: C-strain vaccinated and challenged group (C vacc & chal); full line with black circles: infected group.

Without challenge, neither oral vaccination induced any (or very few) signs of disease (Figure 1). No characteristic lesions were observed during necropsy, except for reactive mesenteric or ileo-caecal lymph nodes in two pigs vaccinated with either the C-strain or CP7_E2alf. Interestingly, a higher blood lymphocyte count was apparent at 12 dpv in the group vaccinated with CP7_E2alf whereas transient lymphocyte depletion was observed at 5 dpv in the group vaccinated with C-strain (Figure 1d).

After challenge at 2 dpv, both oral vaccinations induced partial clinical protection (Figure 1). With the CP7_E2alf vaccination, the mean clinical score was significantly attenuated to 1 at 5-7 dpi (*p* = 0.03) (Figure 1a), the decrease in daily weight gain observed at 7 dpi was reduced, (Figure 1b) and the duration of hyperthermia was shortened to only one day (on 5 dpi) (Figure 1c). However, the CP7_E2alf vaccine, unlike the C-strain vaccine, did not prevent hyperthermia. No cyanosis was observed at necropsy with either vaccination whereas this typical skin lesion was observed in two infected pigs. Thymus atrophy was observed in only one pig in each vaccinated group but in three infected pigs. Moreover, the duration of lymphopenia was shorter in both vaccinated groups (from 3 to 7 dpi) (Figure 1d), and significantly attenuated in comparison to the infected group between 5 and 14 dpi (*p* < 0.01). Blood lymphocyte counts were increased in both vaccinated groups and attained higher levels than the control group at 18 dpi (*p* < 0.05), whereas this was not observed with the infection alone. In this experiment, only one pig (No. 3906) in the infected group did not recover from the disease and had to be euthanized prematurely at 21 dpi. All vaccinated pigs had recovered by the second week post-challenge, whereas two unvaccinated pigs still presented signs of disease.

### CP7_E2alf vaccine reduces viremia duration

Without challenge, the CP7_E2alf vaccine genome was rarely detected by rRT-PCR in blood (one pig from 7 to 9 dpv and another at 5 dpv only) and the C-strain vaccine genome was detected in all pigs from 9 to 12 dpv only (data not shown). After challenge at 2 dpv, virus isolation was delayed in both vaccinated groups. No CP7_E2alf vaccinated pig was positive, one C-strain vaccinated pig was positive whereas four unvaccinated pigs were positive at 3 dpi (Table 1). Although the CSFV genome was detected in all infected groups throughout the experiment, it was not detected in one CP7_E2alf vaccinated pig or in three C-strain vaccinated pigs at 25 dpi. In addition to this slight reduction of the duration of viremia with both vaccines, a maximum mean titer of 10^3.0^ TCID_50_/mL was detected at 5 dpi in pigs vaccinated with CP7_E2alf and of 10^4.1^ TCID_50_/mL at 7 dpi in unvaccinated pigs.

**Table 1:** **CSFV genome detection using real time RT**-**PCR and virus titration in blood from infected groups**

**Group**	**Pig no**	**2**	**5**	**7**	**9**	**12**	**14**	**16**	**20**	**23**	**27**	**Dpv**
		**0**	**3**	**5**	**7**	**10**	**12**	**14**	**18**	**21**	**25**	**Dpi**
infected	3906	-/NI	**+/1.7**	**+/3.5**	**+/4.7**	**+/5.7**	**+/6.5**	**+/5.7**	**+/5.9**	**+/6.1**	na	
	3920	-/NI	**+/1.7**	**+/3.5**	**+/4.3**	+/NI	+/NI	+/NI	+/NI	+/NI	+/NI	
	3912	-/NI	**+/2.1**	**+/3.1**	**+/3.3**	+/NI	+/NI	+/NI	+/NI	+/NI	+/NI	
	3934	-/NI	**+/1.9**	**+/3.1**	**+/4.5**	**+/4.1**	**+/3.5**	**+/2.9**	+/NI	+/NI	+/NI	
	3935	-/NI	+/NI	**+/3.7**	**+/3.9**	**+/1.7**	+/NI	+/NI	+/NI	+/NI	+/NI	
CP7_E2alf vacc & chal	3917	-/NI	+/NI	**+/2.9**	+/NI	+/NI	+/NI	+/NI	+/NI	-/NI	-/NI	
	3902	-/NI	+/NI	**+/3.3**	**+/3.5**	+/NI	+/NI	+/NI	+/NI	+/NI	+/NI	
	3907	-/NI	+/NI	**+/3.3**	**+/4.7**	**+/3.9**	**+/1.9**	+/NI	+/NI	+/NI	+/NI	
	3931	-/NI	+/NI	**+/2.5**	+/NI	+/NI	+/NI	+/NI	+/NI	+/NI	+/NI	
	3911	-/NI	+/NI	**+/2.9**	**+/2.9**	+/NI	+/NI	+/NI	+/NI	+/NI	+/NI	
C-strain vacc & chal	3918	-/NI	+/NI	**+/2.1**	+/NI	+/NI	+/NI	+/NI	-/NI	-/NI	-/NI	
	3933	-/NI	+/NI	+/NI	**+/1.7**	**+/1.7**	+/NI	+/NI	+/NI	-/NI	-/NI	
	3915	-/NI	+/NI	**+/2.9**	**+/2.7**	+/NI	+/NI	+/NI	+/NI	+/NI	-/NI	
	3905	-/NI	+/NI	**+/3.3**	**+/4.1**	**+/3.7**	**+/2.7**	**+/2.3**	+/NI	+/NI	+/NI	
	3903	-/NI	**+/1.7**	**+/3.1**	**+/3.7**	**+/1.7**	+/NI	+/NI	+/NI	+/NI	+/NI	

Plus sign indicates positive genome detection (Ct ≤ 45). Minus sign indicates negative genome detection (Ct > 45). Virus titre values are given for 10^(x)^ TCID_50_/mL of blood. NI indicates that virus were not isolated. Double positive results are shown in bold type. Double negative results are underlined. na: sample not available because of death of the pig; CP7 vacc & chal: CP7_E2alf vaccinated and challenged; C vacc & chal: C-strain vaccinated and challenged; dpv: day post-vaccination; dpi: day post-infection or challenge.

### CP7_E2alf vaccine attenuates the modulations of cytokine production related to CSFV infection

**Figure 2:**
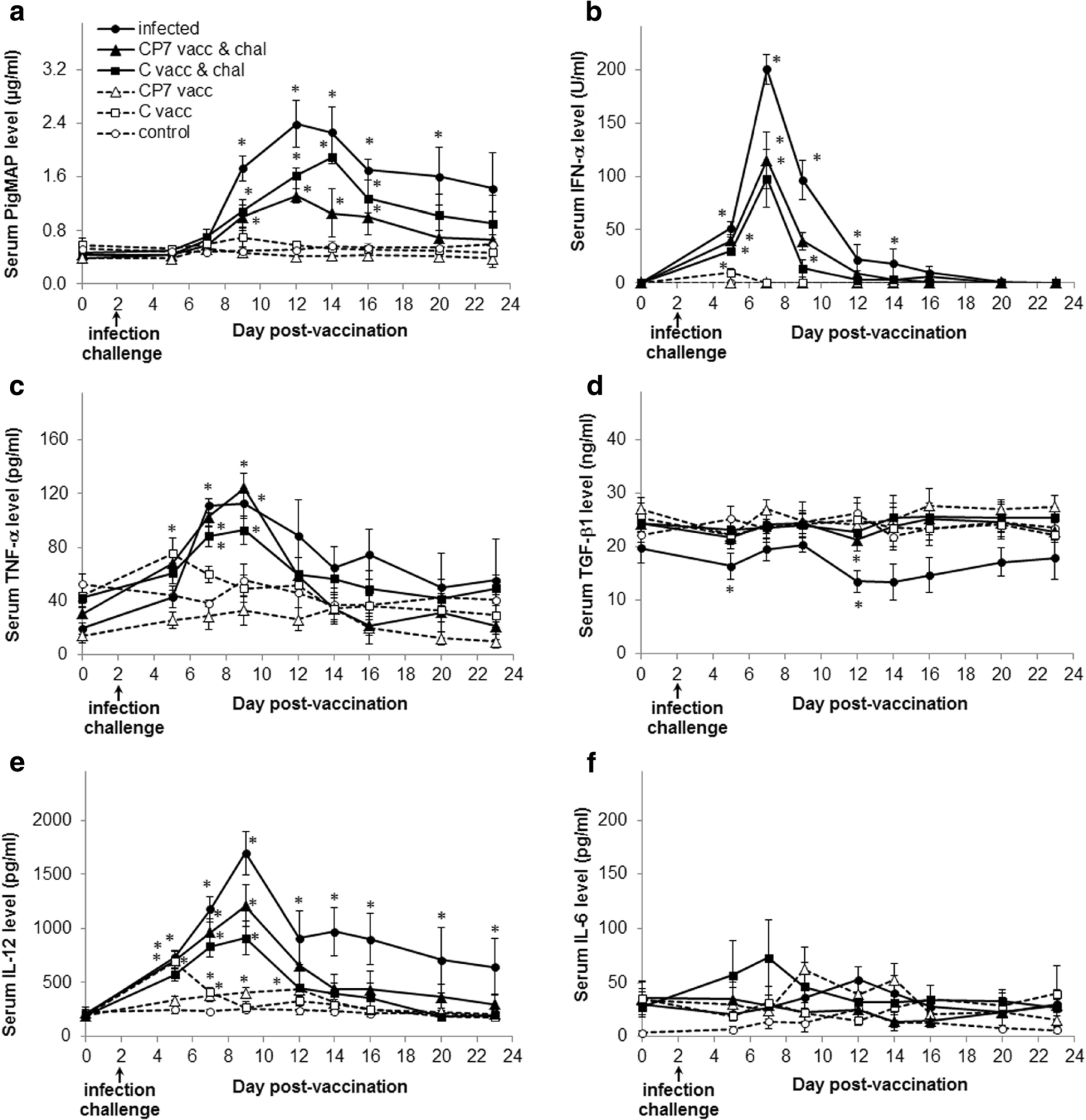
**Cytokine profiles in serum from experimental groups.** Levels were quantified using ELISA for (**a**) PigMAP, (**b**) IFN-α, (**c**) TNF-α, (**d**) TGF-β1, (**e**) IL-12 and (**f**) IL-6. All data are reported as the mean (± SE) of results obtained for the 5 pigs in each group. *p < 0.05 compared each post-vaccination, post-challenge or post-infection value with the respective control value using the Mann–Whitney test. Dotted line with white triangles: CP7_E2alf vaccinated group (CP7 vacc); dotted line with white squares: C-strain vaccinated group (C vacc); dotted line with white circles: control group; full line with black triangles: CP7_E2alf vaccinated and challenged group (CP7 vacc & chal); full line with black squares: C-strain vaccinated and challenged group (C vacc & chal); full line with black circles: infected group.

Without challenge, and compared to the control pigs, the serum levels of PigMAP, TGF-β1 and IL-6 were not significantly modified by either vaccination (Figure 2a, 2d, 2f). No differences in IFN-α and TNF-α levels were observed after vaccination with CP7_E2alf. In contrast, C-strain vaccination produced low levels of IFN-α at 5 dpv (Figure 2b) and a slight increase in TNF-α at 5 dpv (Figure 2c). Although the IL-12 levels induced by both vaccinations were significantly higher than in the controls, the increase occurred later and was smaller with CP7_E2alf vaccination than with C-strain vaccination, with maximum values of 434 pg/mL at 12 dpv and 693 pg/mL at 5 dpv, respectively (Figure 2e).

After challenge at 2 dpv, the duration of high levels of infection-induced PigMAP was reduced to 14 dpi with both vaccines, compared to unvaccinated pigs (Figure 2a). In general, the PigMAP levels at 12 dpi were decreased by CP7_E2alf vaccination, as compared to the infected group without vaccination (*p* < 0.01). Likewise, the production of infection-induced IFN-α was shortened from 12 to 7 dpi and the maximum level at 5 dpi was significantly reduced with both vaccines (*p* < 0.02) (Figure 2b). No significant modulation of TNF-α production was obtained post-challenge between vaccinated and unvaccinated animals. A slightly lower TNF-α level was measured on 14 dpi in CP7_E2alf vaccinated pigs as compared to unvaccinated pigs (Figure 2c). Due to the high variability in TNF-α levels between groups at the beginning of the experiment, bias was avoided by also applying the Wilcoxon test to statistically compare each after-treatment value with the before-treatment (0 dpv) value. The duration of high levels of TNF-α was significantly reduced in both vaccinated groups (*p* = 0.04). CSFV infection led to a decrease in TGF-β1 levels at 10 dpi (14 ng/mL), which was reduced by CP7_E2alf vaccination (*p* = 0.028) (21 ng/mL) and prevented by C-strain vaccination (*p* = 0.47) (23 ng/mL) (Figure 2d). These vaccination-induced differences, compared with the infected group, were significantly extended at 14 and 18 dpi with the CP7_E2alf vaccine (*p* = 0.047) and at 12, 14 and 18 dpi with the C-strain vaccine (*p* = 0.047). The increased level of cytokine IL-12 induced by Bas-Rhin infection attained 1698 pg/mL at 7 dpi and remained significant until the end of the experiment (*p* = 0.016) (Figure 2e). Compared with the infected group, the CP7_E2alf vaccination did not significantly attenuate infection-induced IL-12 production (*p* = 0.175) but differences indicating an earlier return of the IL-12 levels to normal were apparent at 12 and 14 dpi (*p* = 0.047). With C-strain vaccination, the increase in infection-induced IL-12 production was reduced throughout the experiment (at 3, 7, 12, 14, 18 and 21 dpi) (*p* < 0.05). No significant modulation of IL-6 production in serum was obtained either post-infection or post-vaccination (Figure 2f).

**Figure 3:**
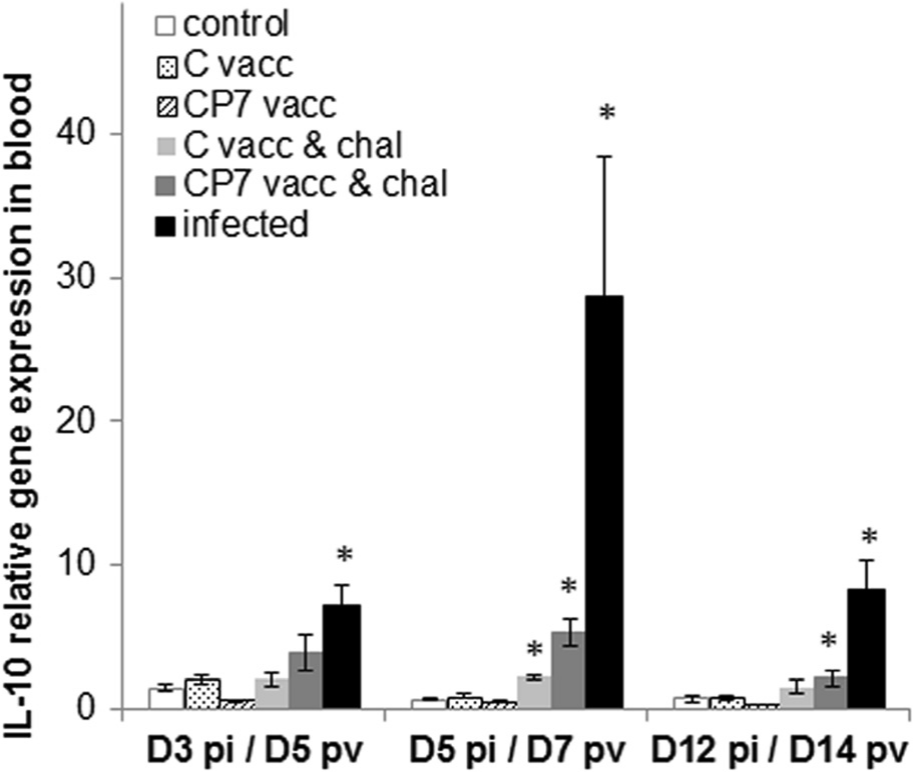
**IL-10 gene expression assessed using real-time RT-PCR in blood from experimental groups.** The values presented are the relative expressions calculated from the Ct results on D3, D5 and D12 post-infection (respectively D5, D7 and D14 post-vaccination), in relation to D0 as described in Materials and Methods. All data are reported as the mean (± SE) of results obtained for the 5 pigs in each group. *p < 0.05 compared each post-vaccination, post-challenge or post-infection value with the respective control value using the Mann–Whitney test. CP7 vacc: CP7_E2alf vaccinated; C vacc: C-strain vaccinated; CP7 vacc & chal: CP7_E2alf vaccinated and challenged; C vacc & chal: C-strain vaccinated and challenged.

The proteins IL-1β, IL-18 and IL-10 were also assessed by ELISA, but could not be quantified in serum from any of the pigs, with similar values to the background values. Although the IL-10 protein was not detected in serum, expression of the IL-10 gene was measured in unpurified blood cells using rRT-PCR. Without challenge, gene expression was similar to that of control pigs after both types of vaccination (Figure 3). After challenge at 2 dpv, IL-10 gene expression induced by the Bas-Rhin infection was significantly attenuated in both vaccinated groups, usually at 5 dpi, compared to the unvaccinated control group (*p* = 0.009).

CP7_E2alf vaccine stimulates IgG2 production post-challenge and differences from the C-strain vaccine were observed for neutralizing antibodies (NAb) and IgG1 production without challenge.

**Figure 4:**
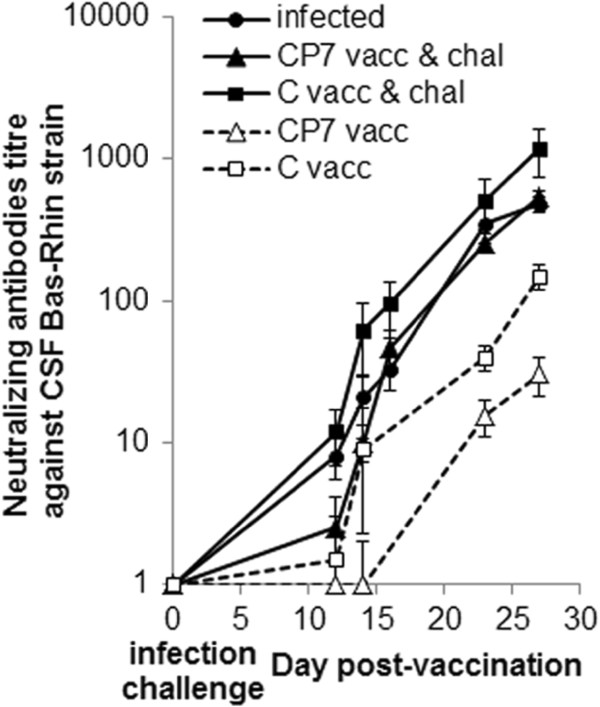
**Neutralizing antibody response in serum from experimental groups.** Results of the neutralizing assay against the Bas-Rhin CSFV strain for vaccinated, challenged and infected groups. All data are reported as the mean (± SE) of results obtained for the 5 pigs in each group. *p < 0.05 compared each post-challenge value with the respective post-infection value using the Mann–Whitney test. Dotted line with white triangles: CP7_E2alf vaccinated group (CP7 vacc); dotted line with white squares: C-strain vaccinated group (C vacc); full line with black triangles: CP7_E2alf vaccinated and challenged group (CP7 vacc & chal); full line with black squares: C-strain vaccinated and challenged group (C vacc & chal); full line with black circles: infected group.

**Figure 5:**
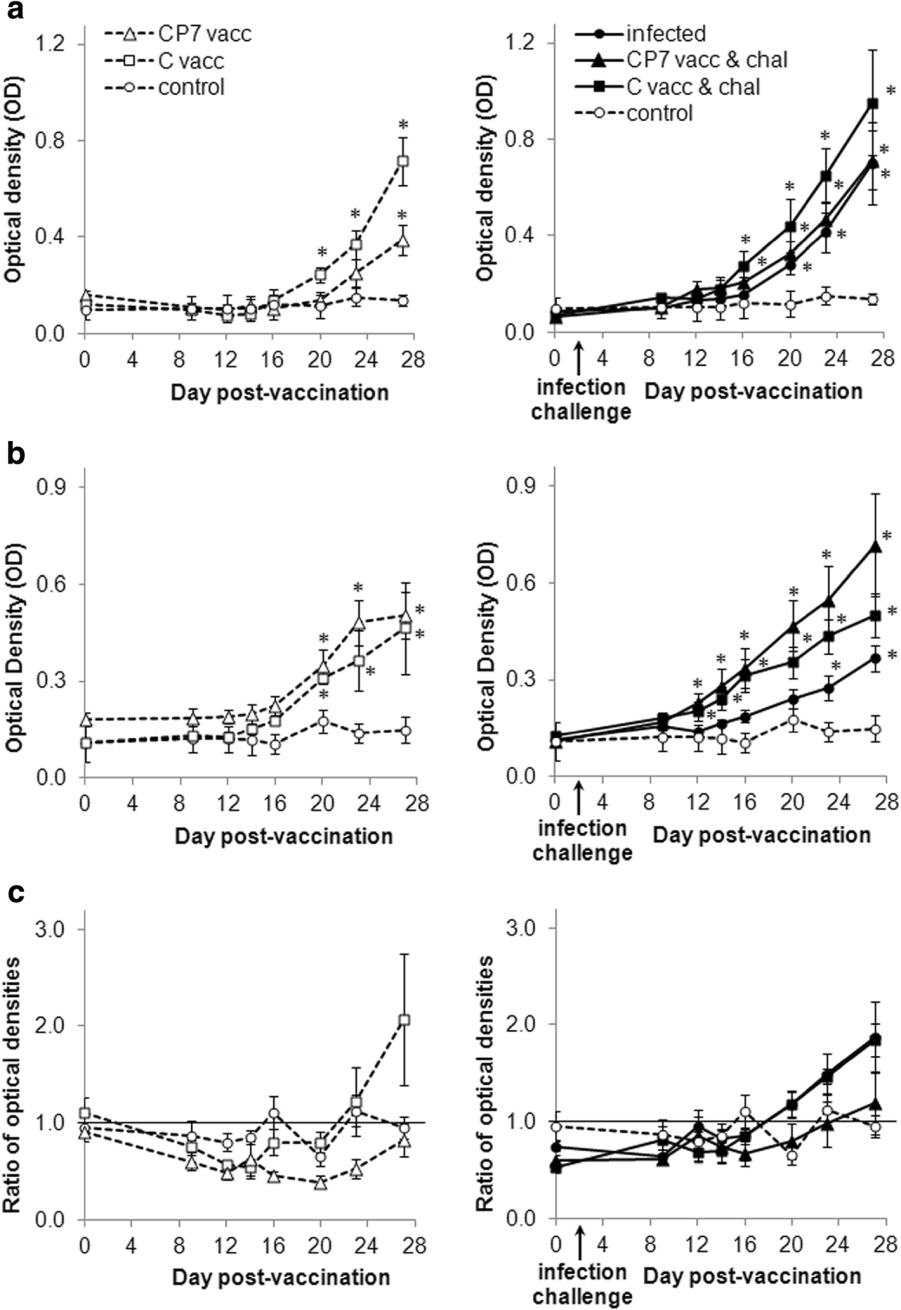
**CSFV**-**specific IgG responses with IgG subclass differentiation assessed by ELISA in serum from experimental groups. a**) Results for the IgG1 subclass. (**b**) Results for the IgG2 subclass. (**c**) Results for the IgG1:IgG2 ratio. All samples were assessed the same day with positive and negative controls on each plate in order to check the sensitivity and the reproducibility of the assay. All data are reported as the mean (± SE) of OD with subtracted blank value, obtained for the 5 pigs in each group. *p < 0.05 compared each post-vaccination, post-challenge or post-infection value with its respective control value using the Mann–Whitney test. Dotted line with white triangles: CP7_E2alf vaccinated group (CP7 vacc); dotted line with white squares: C-strain vaccinated group (C vacc); dotted line with white circles: control group; full line with black triangles: CP7_E2alf vaccinated and challenged group (CP7 vacc & chal); full line with black squares: C-strain vaccinated and challenged group (C vacc & chal); full line with black circles: infected group.

Without challenge, the C-strain vaccine induced a higher production of NAb against the Bas-Rhin strain than the CP7_E2alf vaccine at 14, 23 and 27 dpv (*p* > 0.05), with titres of 150 and 31 at 27 dpv (Figure 4). After challenge at 2 dpv, NAb against the Bas-Rhin strain were apparent from 12 dpv (10 dpi) in the group vaccinated with C-strain and from 14 dpv (12 dpi) in the group vaccinated with CP7_E2 (Figure 4). However the differences between the vaccinated and unvaccinated groups were never significant. Both major sub-classes of IgG anti-CSFV antibodies were detected in the serum. The IgG1 profile was similar to the above-described NAb profile, the C-strain vaccine inducing more IgG1 production than the CP7_E2alf vaccine at 20 and 27 dpv without challenge (*p* < 0.05) and no difference with either vaccine post-challenge (Figure 5a). Conversely, without challenge, no differences between the two vaccines were detected (*p* > 0.05) and post-challenge, both vaccines induced higher IgG2 levels than the unvaccinated group as of 10 dpi (Figure 5b). From these results, the IgG1:IgG2 ratio was calculated to get information about the specific immune response potentiation; the ratio value was lower (Th1) or higher (Th2) than 1. Interestingly, without challenge, a low ratio was observed with CP7_E2alf vaccine at all days pv whereas a high ratio was observed with C-strain vaccine at 23 dpv (ratio = 1.22) and 27 dpv (ratio = 2.07) (Figure 5c). Post-challenge, a single high ratio was obtained at 25 dpi (27 dpv) with the CP7_E2alf vaccine whereas high ratios were obtained with the C-strain from 18 dpi (20 dpv) onwards (Figure 5c).

We then examined the hypothesis that the CP7_E2alf vaccine induced a slightly different specific immune response by assessing the Th1 and Th2 response-related cytokines IFN-γ and IL-4. However, the values obtained for these cytokines were below the limits of detection both in serum by ELISA or in unpurified blood cells by relative quantitative rRT-PCR (data not shown).

## Discussion

This paper demonstrates the similar abilities of the CP7_E2alf marker vaccine and the C-strain vaccine to confer partial protection against CSFV challenge with a recent MV isolate, as early as 2 days post-oral immunization. To our knowledge, this is the first study to investigate the efficacy of CP7_E2alf vaccine less than one week post-immunization. An early onset of partial protection against a challenge with MV strains had already been reported 3 days after intramuscular vaccination with the C-strain vaccine and complete protection after 5 days [12]. Effective protection occurred more rapidly when the CP7_E2alf vaccine was administered by the intramuscular route than by the oral route [13]. This study also provides further evidence of the early efficacy of live vaccines administered by oral route and supports that the CP7_E2alf vaccine could be a good alternative to the C-strain vaccine for the emergency vaccination of pigs or for oral vaccination campaigns in wild boar.

Both vaccines attenuated pathogeny to a similar extent and before the appearance of NAb. During CSF, viremia is generally highly dependent on serum NAb levels. Our results show that viremia decreased from 7 dpi onwards in all groups and was already well advanced when NAb was detected in the serum at 12 dpi (14 dpv). Moreover, the NAb titers did not differ significantly between vaccinated and unvaccinated groups, suggesting that other immune responses, in addition to the NAb response, are involved in the early onset of partial protection. The immune responses induced by live vaccines to provide rapid protection against CSFV infection, and particularly the role of cytokines, are not fully understood. Our results show that both live vaccines were able to attenuate some of the cytokine responses induced by CSFV infection. For example, IFN-α may be involved in CSFV pathogeny and particularly in lymphopenia induction [16]. In our study, vaccination was found to reduce serum IFN-α levels post-infection. Vaccination with DNA vaccine expressing the E2 CSFV protein has already been shown to attenuate the IFN-α level [25]. Surprisingly, in the absence of challenge, a low IFN-α level in relation to the low lymphopenia observed at 5 dpv, was only induced by C-strain vaccine, in contrast to the higher attenuation of IFN-α level observed post-challenge. The transient IFN-α level induced by C-strain vaccination may stimulate an early host immune response to control the production of IFN-α induced by CSFV infection. During CSFV infection, high levels of proinflammatory cytokines, suspected to be associated with the hemorrhage characteristic of the acute form of the disease, are released by the macrophages [17,26]. Our results show that TNF-α production was not significantly affected by vaccination and that neither IL-1 nor IL-6 could be detected. We previously reported the lower production of TNF-α induced by infection with a MV strain of CSFV, and suggested that, in contrast to HV strains, TNF-α may be of secondary importance with MV strains [27]. Few hemorrhagic lesions are produced in the sub-acute form of the disease observed after infection with an MV strain, which suggests that a lack of proinflammatory cytokines is released.

Acute phase proteins are good indicators of the inflammatory reactions caused by infection, injury or stress and can be used to monitor animal health. The production of these proteins in the liver is mainly controlled by proinflammatory cytokines [28]. An acute phase response has already been observed in pigs inoculated with CSFV [29]. In this study, CSFV infection produced an increase in PigMAP, which was attenuated by vaccination. Discrimination between vaccinated and unvaccinated animals, based on serum PigMAP levels, has already been demonstrated after infection with the Aujeszky’s disease virus [30].

IL-10 levels are increased post-infection and this cytokine is assumed to have a key role in immunosuppression observed during the course of CSF disease [17,31]. Further support for this key role was provided by the attenuation of IL-10 gene expression induced by both vaccines following CSFV challenge. Conversely, the production of another immunosuppressive mediator, TGF-β1, was repressed after infection with an MV strain, and then prevented by vaccination. Our results confirm those of a previous study involving microarray analysis that showed that TGF-β was down-regulated as early as 1 dpi in PBMC isolated from pigs infected with an HV strain [32]. TGF-β controls inflammation and auto-aggressive immune reactions by CD4+CD25+ regulatory T cell activation [33]. The impaired TGF-β1 repression observed in CSFV infection post-vaccination suggests that this effect may also be related to maintenance of the inflammatory condition and high levels of cytokines. Interestingly, the levels of IL-12 were enhanced in response to CSFV infection and then attenuated by the vaccination. A relationship was observed between the severity of clinical signs and the IL-12 levels, as illustrated by the one infected pig which displayed a continual increase in IL-12 levels until death. IL-12 is produced by dendritic cells in the blood and spleen after CSFV infection [17] and co-delivery of IL-12 with a DNA vaccine reduces both the NAb titres and protection against a CSFV challenge [34]. Our results, however, are the first real element suggesting involvement of IL-12 in CSFV pathogeny. IL-12 may play a pathogenic role by enhancing the chemoattraction of activated macrophages induced by the CSFV infection thereby promoting virus replication and leading to high levels of proinflammatory cytokines [35].

Thus, the observed post-infection modulation of the responses of cytokines and their attenuation by vaccination suggest that these changes in cytokine levels are related to CSFV pathogeny rather than to protection. It would now be interesting to understand the CSFV-induced mechanisms which generate these cytokine modulations and how live vaccines interfere with these modulations. One explanation of the interference of vaccines, in relation to pathogeny attenuation or partial protection, could be that the vaccine and challenge strains compete for replication sites in the tonsils. This hypothesis is supported by the longer delay for isolation of the virus in blood from vaccinated, as compared to unvaccinated, pigs.

The IL-12 produced by dendritic cells promotes IFN-γ secretion by the T cells and NK cells. Together IL-12 and IL-18 play a synergistic role in this activation [36]. However, no production of IFN-γ or IL-18 was detected in this study. During infection, IL-10 facilitates immunosuppression and viral persistence by inhibiting the activity of Th1 and NK cells [37,38]. Thus, the absence of IFN-γ detection may be due to the enhancement of IL-10 mediated by CSFV. Previous studies have shown that the C-strain vaccine induces IFN-γ production post-vaccination [12,39]. In our study, the absence of IFN-γ detection was probably due to poor sensitivity of the assay. The humoral immune response plays a major role in protection against CSFV. IL-4 is produced by Th2 cells and stimulates B cell activation and immunoglobulin-class switching [40] and is considered as one of the main indicators of antibody-mediated immunity. However, IL-4 was not detected in this study even though high titres of neutralizing antibodies were measured, thereby confirming that serum may not be the best biological matrix for IL-4 or IFN-γ assays [41]. Also, in many other studies the intramuscular route was used and it may not be possible to detect these cytokines after immunization by the oral route.

In swine, IFN-γ has been shown to be related to IgG2 production [42] which supports the hypothesis that the IgG1:IgG2 ratio could provide a suitable indicator of adaptive immunity potentiation towards a Th1 or Th2 response, as in other species. Interestingly, our results showed that the post-challenge production of CSFV-specific IgG2, but not IgG1, was enhanced in vaccinated as compared to unvaccinated pigs. This suggests that IgG2, and possibly the Th1 immune response, have a role in immunity against CSFV infection. Surprisingly, in relation to the NAb response, vaccination with the C-strain and without challenge seemed to stimulate IgG1 production to a greater extent than CP7_E2alf vaccination. The high IgG1:IgG2 ratio clarified this predominance of Th2-humoral response in animals vaccinated with the C-strain vaccine compared with CP7_E2alf vaccinated animals, which would counterbalance towards Th1-cellular response. Nevertheless this possible difference in trend between the vaccines could not be correlated with the IFN-γ and IL-4 levels, due to non-detection of these proteins, and therefore remains hypothetical. However, without challenge, the lymphocyte counts increased earlier in blood after CP7_E2alf vaccination than after C-strain vaccination (from 12 dpv and 20 dpv, respectively), suggesting that stimulation of lymphocyte proliferation was improved with the CP7_E2alf vaccine. Further investigations need to be carried out to identify the lymphocyte sub-populations concerned and to bring results supporting different specific immune responses induced by the two vaccines.

To conclude, the similar early onset of partial protection induced after oral immunization with the marker vaccine CP7_E2alf demonstrated its powerful potential as a substitute for the conventional C-strain vaccine. The interference of both vaccines in the post-CSFV infection responses of cytokines IFN-α, PigMAP, TGF-β1, IL-12 and IL-10 supports that the production of these cytokines is related to CSFV pathogeny. We also observed that CSFV-specific IgG2 production was enhanced by both vaccines, suggesting that the Th1-immune response may have a role in protection against CSFV infection. Differences between the C-strain and CP7_E2alf vaccines, with regards to the balance of induced adaptive immune responses, are hypothesized but require further investigation.

## Competing interests

The authors declare that they have no competing interests.

## Authors’ contributions

PR conceived and coordinated the experiment, performed cytokine quantifications, statistical tests, analyzed data and drafted the manuscript. MLD performed virological tests, serological tests and cytokine quantifications. AK and RC monitored clinical signs and supervised the experiment. FK conceived and coordinated the CSF_goDIVA project and reviewed the manuscript. MFLP supervised the study and reviewed the manuscript. All the authors read and approved the final manuscript.
